# A Holistic Digital Health Framework to Support Health Prevention Strategies in the First 1000 Days

**DOI:** 10.2196/55235

**Published:** 2025-01-16

**Authors:** Silvia Gabrielli, Oscar Mayora Ibarra, Stefano Forti

**Affiliations:** 1 Digital Health Research, Fondazione Bruno Kessler, Via Sommarive 18, Trento, 38123, Italy, 39 0461 312 477; 2 Center for Digital Health & Wellbeing, Fondazione Bruno Kessler, Trento, Italy

**Keywords:** digital health, digital therapeutics, behavioral intervention technology, prevention, citizen science, first 1000 days

## Abstract

The first 1000 days of a child’s life, spanning from the time of conception until 2 years of age, are a key period of laying down the foundations of optimum health, growth, and development across the lifespan. Although the role of health prevention programs targeting families and children in the first 1000 days of life is well recognized, investments in this key period are scarce, and the provision of adequate health care services is insufficient. The aim of this viewpoint is to provide a holistic digital health framework cocreated with policy makers, health care professionals, and families to support more effective efforts and health care programs dedicated to the first 1000 days of life as the first line of prevention. The framework provides recommendations for leveraging on behavioral intervention technology and digital therapeutics solutions augmented by artificial intelligence to support the effective deployment of health prevention programs to families. The framework also encourages the adoption of a citizen science approach to co-design and evolve the digital health interventions with all relevant stakeholders in a real-world research perspective.

## Introduction

The first 1000 days is a continuum that begins with pregnancy and ends at the child’s second birthday. It is a unique period laying down the foundations of optimum health, growth, and development across the lifespan [1], but it can also represent a period of potential vulnerability where the way mothers and children are cared for has a profound influence on a child’s ability to grow, learn, and thrive [2]. Although the role of the first 1000 days of life is well recognized [2,3], investments in this key period are scarce, and the provision of adequate health care services and interventions is insufficient [1].

In this viewpoint, we address current challenges and opportunities in the development of effective health care interventions for the first 1000 days, by leveraging on a holistic digital health (DH) framework that can help to optimize efforts in the cocreation of these interventions with the support of policy makers, health care professionals, and families. The framework leverages on state-of-the-art approaches and opportunities in the design of behavioral intervention technology (BIT) and artificial intelligence (AI)–augmented digital therapeutics (DTx) for prevention and care, such as the Integrate, Design, Assess, and Share (IDEAS) framework [4] and the DTx Real-World Evidence (RWE) framework [5]. It complements these approaches by stressing the importance of facing the design and validation challenges within a longitudinal perspective based on citizen science and real-world evidence, to cocreate and evolve the DH interventions in a more pragmatic and sustainable way.

## Health Prevention Strategies and Challenges in the First 1000 Days

The first 1000 days are characterized by 3 main periods of intervention, preconception, pregnancy, and infancy, which are key for ensuring children’s healthy growth [1,3]. Health prevention programs and strategies during the preconception period address biomedical, behavioral, and social risks factors that may affect a pregnant woman’s health, by providing nutritional and physiological support; identification and prevention of risks, such as toxic exposures [6,7]; and support in the adoption of changes in lifestyle [3]. Health prevention programs and strategies should also be maintained in the interconception period, going from childbirth until the birth of a subsequent child [8]. During pregnancy, the main areas of prevention typically regard nutrition, stress, and exposure to environmental contaminants [3,9]. The Italian Ministry of Health identifies 11 thematic areas that focus on prevention in the first 1000 days, including nutrition, lifestyle, parental literacy and skills, and mental health [10].

In different countries, public health care programs and interventions have been developed to support families in the first 2 years after birth [11-14], promoting health literacy and behavioral change of parents in key areas such as nutrition, lifestyles, and mental health. Notwithstanding the large amount of data and guidelines supporting the importance of establishing efficient health care services in the first 1000 days, these health prevention strategies have not been efficiently converted into comprehensive and integrated programs enabling adequate support to parents and infants during this period [1,15,16].

In this viewpoint, we advocate that this gap is also due to a lack of deployment of DH solutions that support evidence-based educational and behavioral interventions for families and are designed for being acceptable, inclusive, engaging, equitable, scalable, and sustainable over the lifespan. Recent reviews of health professional-delivered interventions during the first 1000 days have shown that so far, most interventions were delivered in individual or group face-to-face sessions and that optimal intervention, in terms of timing, content, dose, mode of delivery, theory, and active ingredient, have yet to be established [15].

In our vision, DH technologies can play a key role in supporting the deployment of health prevention strategies and programs in the first 1000 days (as the first line of prevention) as well as over the individuals’ lifespan. The kind of automation, engagement, and decisions supported by DH empowered by AI are commonly reviewed by domain experts before they can be implemented in a treatment plan, a process called augmented intelligence or intelligence amplification [17], wherein AI technology informs and augments, rather than replaces, health care professionals’ experience and cognition [18].

**Figure 1.**
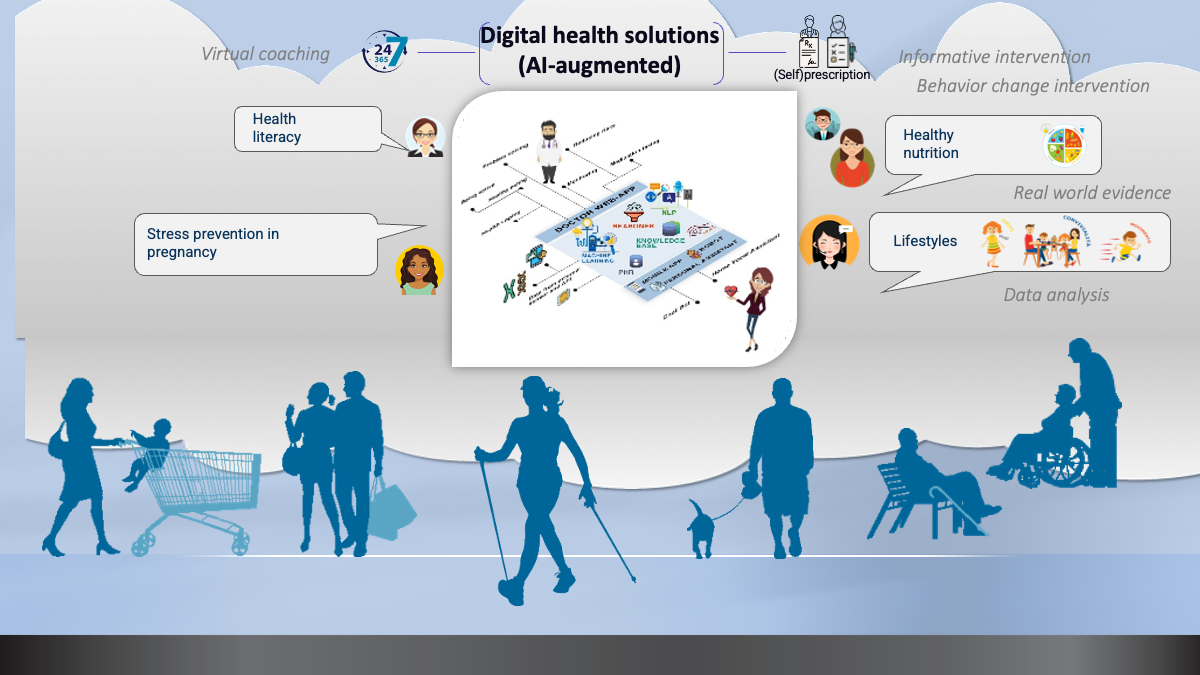
Digital health solutions delivering prevention programs in the first 1000 days and over the lifespan. AI: artificial intelligence.

As depicted in Figure 1, prevention programs addressing key areas of intervention, such as nutrition, lifestyle, mental health and well-being, health literacy, and education, may be more effectively translated into behavioral intervention solutions. These interventions should be informed by evidence-based theoretical approaches and a diversity of potential technologies to ensure a scalable, sustainable, accessible, and cost-effective delivery of prevention strategies. To address the challenges of effectively designing such solutions we call for a holistic DH framework helping to combine behavioral, technical, and methodological components in the intervention design process to ensure a translation of health strategies into better prevention and health outcomes for the target populations.

## A Holistic DH Framework for Cocreation With Stakeholders

### Opportunities and Challenges of DH Deployment

Recent advances in the development of DH solutions provide unprecedented opportunities for deploying health prevention strategies and programs with the support of BITs or DTx. BITs typically include sensor-trackers (eg, heart rate and step count), AI-augmented chatbots (automated conversational agents), and momentary ecological assessments (which can repeatedly assess individual’s behaviors and experience them in real time) [19-21].

DTx are defined as tools to deliver evidence-based health interventions (using, for example, cognitive behavioral theory as an active ingredient for treatment) through different types of potential technological solutions as excipients (eg, mobile health apps, web apps, chatbots, or virtual reality [VR] environments) [22-24].

Both BITs and DTx are DH solutions that can improve health outcomes, reduce burdens on health care professionals, and increase access to and usability of interventions [25,26]. Common goals of DH include improving lifestyle, by facilitating behavior change in diet, physical activity, or sleep, or improving mental health, such as care for depression, anxiety, or symptoms of stress. DH solutions are often complex interventions [27], as they can include multiple components, such as goal-setting or problem-solving elements and AI algorithms that adapt provision of support to each person’s changing needs. The goal of including these components in DH solutions is that they can simultaneously provide safe, effective, accessible, sustainable, scalable, and equitable support for individual and population health [28]. However, accomplishing an effective integration of these components in DH solutions is very difficult, and recent efforts have been made to provide guidance in the design, development, and assessment of these solutions by leveraging on the evidence offered by real-world data produced over usage of these digital tools, to better understand the trajectories of intervention outcomes over time [5].

### The Framework Development

**Figure 2.**
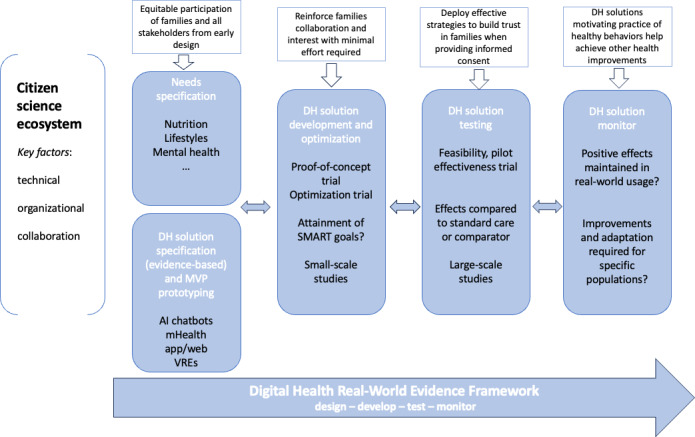
Holistic DH framework to cocreate with stakeholders. AI: artificial intelligence; DH: digital health; mHealth: mobile health; MVP: minimum viable product; SMART: Specific, Measurable, Actionable, Realistic, Timely; VRE: virtual reality environment.

The holistic DH framework (Figure 2) is aimed at supporting the future deployment of DH solutions for prevention in the first 1000 days.

The authors of this paper first conducted a review of state-of-the-art frameworks relevant to inform the design and validation of these solutions. The review identified strengths and limitations of 4 main frameworks commonly deployed in the design of digital interventions: (1) the IDEAS framework, aimed at supporting the development of digital interventions for health behavior change and based on the IDEAS phases [4]; (2) the ORBIT (Obesity-Related Behavioral Intervention Trials) model [29], aimed at guiding the design of evidence-based behavioral treatments to prevent and treat chronic diseases, based on the 4 phases of design, preliminary testing, efficacy, and effectiveness research; (3) the MOST (Multiphase Optimization Strategy) model [30,31], aimed at optimizing the development of behavioral, biobehavioral, and biomedical interventions; and (4) the DTx RWE framework [5], inspired by ORBIT and based on the 4 phases model of development (design, develop, test, and monitor). The review also included the citizen science approach [32] as a relevant method informing the cocreation of DH solutions with the participation of all stakeholders. Citizen science has been defined as the general public engagement in scientific research activities, when citizens actively contribute to science either with their intellectual effort or surrounding knowledge or their tools and resources [33].

The holistic DH framework was then developed in a 2-way process. First, 1 author (SG) selected the combination of components from the DTx RWE framework and citizen science approach most relevant to provide a comprehensive overview of the research steps to be taken to design and deploy digital interventions for health prevention programs. In a second step, a draft version of the framework was discussed at length in a meeting among the 3 authors (SG, OMI, and SF) and revised based on the feedback provided.

We present the key components of the holistic framework in a case study providing guidance and recommendations for ensuring a more effective deployment of health prevention programs in the first 1000 days by, at the same time, leveraging on the potential of DH solutions.

## Application of the Holistic DH Framework

**Figure 3.**
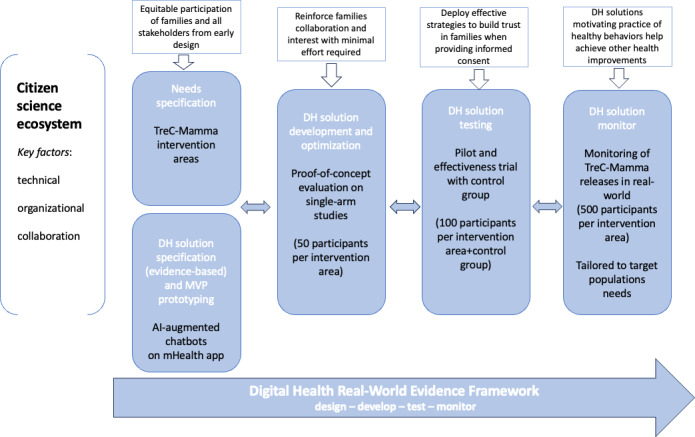
Holistic DH framework applied to *first 1000 days* interventions. AI: artificial intelligence; DH: digital health; mHealth: mobile health; MVP: minimum viable product

To illustrate the potential of the holistic DH framework in guiding the cocreation of effective prevention programs in the first 1000 days, we present examples of ongoing activities started by our research team in tackling the issues of digitizing prevention programs currently available to citizens in the Trentino region (Italy) for the pregnancy and infancy periods (birth path), working together with health professionals (gynecologists, obstetricians, and pediatricians) at the local health care system, with policy makers at the local Department of Health and Innovation, and with the target user populations (families). The main intervention areas addressed so far regard providing parental literacy to women and families during the first 1000 days (Figure 3), and providing pregnant women with healthy nutrition and psychoeducational DH programs for stress prevention from the 4th month of gestation [34-37]. The overall goal of introducing digital tools to support current prevention programs was to facilitate the adoption of a holistic strategy to target barriers in the delivery of these programs, such as reaching women living in rural areas having difficulties in accessing health care services or belonging to vulnerable populations, but also to improve ongoing prevention programs’ development by incorporating holism, precision prevention, timeliness, and cost-effectiveness [19,38].

The design phase for these DH solutions started from a series of participatory sessions with all stakeholder groups (health professionals, policy makers, and families) to achieve an in-depth knowledge of the prevention needs in the target areas and to specify the requirements and prototyping of the DH tools by providing equitable consideration of the different stakeholders’ perspectives, as well as building empathy and acknowledgment of any power differential [32].

From a technological point of view, it was decided to realize a secure and privacy-preserving DH platform providing virtual coaching functionalities by means of AI-augmented digital assistants available on mobile app (or coaching avatars in VR) each specialized for delivering DH intervention in a target prevention area.

Chatbot technology, using AI-augmented tools including machine learning and natural language processing, has been introduced into the health sector to address current health care challenges, such as shortage of health care providers and lack of health care access, showing several positive effects in supporting tailored intervention, which is better able to address users’ needs over a digital treatment [39].

At the current stage of DH solutions development, a chatbot-based mobile app supporting women and families during the first 1000 days, named TreC-Mamma (Digital Health Research and Digital Health Innovation Lab, Fondazione Bruno Kessler), has already been released, and it is in use by more than 1000 users in the region. The aim of the TreC-Mamma solution is to provide an initial platform for collecting real-world evidence on the impact of DH solutions deployment during the first 1000 days, thus informing the development and refinement of more advanced AI-augmented digital assistants for the different intervention areas, by relying on a larger involvement and participation of families in the cocreation process. Parallel research activities are ongoing for assessing the developed proof-of-concept DH solution minimum viable products (MVPs) targeting healthy lifestyles and stress prevention during pregnancy. Specifically, virtual coaching on healthy nutrition and physical activity is provided to pregnant women by using microlearning and motivational modules validated by domain experts. For stress prevention, a chatbot-based solution deploying a digitally adapted version of the Self-Help+ protocol developed by WHO (World Health Organization) [40] has been implemented and preliminarily validated with mental health experts.

Small-scale studies are currently running to assess the MVPs’ usability, acceptance, scalability, etc for further refinement and optimization based on the attainment of benchmark criteria such as the SMART (Specific, Measurable, Actionable, Realistic, Timely) goals [41]. These studies, typically involving small samples of pregnant women and their families, are also contributing to reinforce their collaboration and interest in the research project goals in the long-term, aiming to minimize the effort requested from citizens if they participate in the cocreation process.

Once the development and optimization phase of the digital assistants is completed, these DH solutions will be integrated into the TreC-Mamma platform and will be available for use by families to undergo more large-scale testing in the form of feasibility pilot trials or effectiveness trials. The ultimate goal of this testing phase is to compare the effects of the DH interventions with standard of care in the local health care system, with other relevant comparators, or by means of randomized controlled trials. In preparation of this testing phase, we are already devising an effective strategy to build trust in the project aim and solutions among families that are using the TreC-Mamma platform, facilitating the collection of their informed consent through the platform (asking them to sign a so-called “agreement with the citizen”), and clearly explaining the type of data and intended use of their data for a dynamic improvement of the platform in a citizen science perspective.

In a more long-term view, the TreC-Mamma platform will allow us to monitor the effects of our released DH interventions to assess whether their positive effects are maintained over real-world usage. In this monitoring phase, additional requirements and needs may arise from families and other stakeholders, leading to improvements and further adaptations of our solutions to fulfill evolving needs to address new areas of prevention or specific needs of target populations. As a positive side effect of deploying our DH solutions for motivating families to practice healthy behaviors in the first 1000 days, we foresee the potential achievement of health improvements in other prevention areas (eg, chronic disease prevention, such as gestational diabetes, type II diabetes, and depression) contributing to the realization of more comprehensive prevention goals and strategies.

## Conclusions and Future Work

The holistic framework presented aims to provide guidance in the design and deployment of DH solutions supporting prevention strategies in the first 1000 days. It can be used to realize DH interventions in a more effective and participatory approach, leveraging on the contribution of the different stakeholders. It can help researchers in addressing the complex goal of realizing AI-augmented DH solutions by optimizing the resources available in the design process. This can be achieved by deploying a sociotechnical platform able to facilitate the engagement of citizens in the different stages of the intervention validation, as well as the effective use of real-world data for supporting the evolution and adaptation of the intervention to the target user populations.

Among the strengths of the framework presented is that it sheds light on unprecedented opportunities for deploying DH technologies in the attempt to realize more scalable, sustainable, and cost-effective solutions for prevention. It is also based on a concrete example of ongoing applied research for prevention in the first 1000 days that is supporting the feasibility of the approach proposed. We also believe that the framework illustrated can be of help in designing DH solutions for different application fields targeting health prevention and care with other target populations. By combining real-world evidence with citizen science research, it can help to overcome some limitations of previous DH frameworks like IDEAS by, for example, better supporting multidisciplinary teams’ work beyond the initial intervention design and refinement, providing evidence for the adoption of the most effective behavioral strategies as derived from large-scale deployment of the digital solutions, and showcasing the sustainability of the digital solutions deployment, which may facilitate their more structural adoption and support by policy makers and users in the target communities.

However, there are also some limitations involved in the framework adoption and in its generalization to different contexts. Most of the real-world experience reported in the case study presented regards DH solutions design and development, while key outcomes derived from the test and monitor phases are still lacking. Therefore, more validation data on the framework application in real-world settings are needed, and this objective will be part of our future work. In addition, the AI-augmented features recommended to tailor the coaching of the digital assistants to the user needs require considerable design and development efforts, which may consume more tangible and intangible resources [42]. Key ethical considerations should also be considered when applying AI algorithms and techniques in DH solutions development, including bias minimization, transparency, and users’ privacy and safety protection [43,44].

Notwithstanding these limitations, we think that grounding DH design efforts in a real-world evidence practice informed by a citizen science approach may contribute to establishing more productive dialogues among the stakeholders involved, as well as to facilitating faster innovation outcomes in the implementation of prevention programs.

## Abbreviations

AI: artificial intelligence
BIT: behavioral intervention technology
DH: digital health
DTx: digital therapeutics
IDEAS: Integrate, Design, Assess, and Share
MOST: Multiphase Optimization Strategy
MVP: minimum viable product
ORBIT: Obesity-Related Behavioral Intervention Trials
RWE: Real-World Evidence
SMART: Specific, Measurable, Actionable, Realistic, Timely
VR: virtual reality
WHO: World Health Organization
